# The impact of HER2 phenotype of circulating tumor cells in metastatic breast cancer: a retrospective study in 107 patients

**DOI:** 10.1186/s12885-015-1423-6

**Published:** 2015-05-14

**Authors:** Markus Wallwiener, Andreas Daniel Hartkopf, Sabine Riethdorf, Juliane Nees, Martin Ronald Sprick, Birgitt Schönfisch, Florin-Andrei Taran, Jörg Heil, Christof Sohn, Klaus Pantel, Andreas Trumpp, Andreas Schneeweiss

**Affiliations:** 1 Department of Obstetrics and Gynecology, University of Heidelberg, Im Neuenheimer Feld 440, 69120 Heidelberg, Germany; 2 Department of Obstetrics and Gynecology, University of Tübingen, Calwerstraße 7, 72076 Tübingen, Germany; 3 Department of Tumor Biology, University Medical Center Hamburg-Eppendorf, Martinistraße 52, 20246 Hamburg, Germany; 4 National Center for Tumor Diseases, Im Neuenheimer Feld 460, 69120 Heidelberg, Germany; 5 Heidelberg Institute for Stem Cell Technology and Experimental Medicine (HI-STEM gGmbH), Im Neuenheimer Feld 280, 69120 Heidelberg, Germany; 6 Division of Stem Cells and Cancer, German Cancer Research Center (DKFZ), Im Neuenheimer Feld 280, 69120 Heidelberg, Germany

**Keywords:** Metastatic breast cancer, Circulating tumor cell phenotype, Human epidermal growth factor receptor 2, Treatment response, Survival

## Abstract

**Background:**

In metastatic breast cancer (MBC), antigen profiles of metastatic tissue and primary tumor differ in up to 20 % of patients. Reassessment of predictive markers, including human epidermal growth factor receptor 2 (HER2) expression, might help to optimize MBC treatment. While tissue sampling is invasive and often difficult to repeat, circulating tumor cell (CTC) analysis requires only a blood sample and might provide an easy-to-repeat, real-time “liquid biopsy” approach. The present retrospective study was conducted to compare HER2 expression in primary tumors, metastatic tissue, and circulating tumor cells (CTCs) from MBC patients and to analyze the potential impact of HER2 overexpression by CTCs on progression-free (PFS) and overall survival (OS) in MBC.

**Methods:**

CTC-positive (five or more CTCs/7.5 mL blood; CellSearch®, Janssen Diagnostics) MBC patients starting a new line of systemic treatment were eligible for the study. HER2 status of CTCs was determined by immunofluorescence (CellSearch®). HER2 status of primary (PRIM) and metastatic (MET) tumor tissue was determined by immunohistochemistry. Data were analyzed using descriptive statistics and Kaplan–Meier plots.

**Results:**

One hundred seven patients (median age (range) 57 (33–81) years) were included. 100/107 (93 %) patients were followed-up for a median [95 % confidence interval (CI)] of 28.5 [25.1–40.1] months. Of 37/107 (35 %) CTC-HER2-positive patients only 10 (27 %) were PRIM-HER2-positive. 6/46 (13 %) patients were MET-HER2-positive; only 2/10 (20 %) CTC-HER2-positive patients were MET-HER2-positive. Overall accuracy between CTC-HER2 expression and PRIM-HER2 and MET-HER2 status was 69 % and 74 %, respectively. Kaplan–Meier plots of PFS and OS by CTC-HER2 status revealed significantly longer median [95 % CI] PFS of CTC-HER2-positive versus CTC-HER2-negative patients (7.4 [4.7–13.7] versus 4.34 [3.5–5.9] months; *p* = 0.035). CTC-HER2-positive status showed no significant difference for OS (13.7 [7.7–30.0] versus 8.7 [5.9–15.3] months; *p* = 0.287).

**Conclusions:**

HER2 status can change during the course of breast cancer. CTC phenotyping may serve as an easy-to-perform “liquid biopsy” to reevaluate HER2 status and potentially guide treatment decisions. Further, prospective studies are needed.

## Background

Metastatic breast cancer (MBC) is a heterogeneous disease. The current standard for predicting prognosis in MBC is based on the primary tumor’s biological phenotype, the site of metastasis, and the line of therapy. As MBC treatment evolves towards targeted therapy, the efficacy of novel therapies is also increasingly based on the biological characteristics of the disease. However, these are currently determined using primary tumor tissue (e.g. HER2-status) or by means of sequential metastatic tissue biopsies because breast cancer phenotype may change during disease progression [1, 2]. This bears several limitations: (1) Metastatic tissue may not be available, (2) repeated sampling of metastatic tissue may not be feasible due to increased morbidity, and (3) metastatic breast cancer might be heterogeneous, implying that tissue from a single metastasis obtained at a single time point may not adequately reflect the tumor burden.

Currently, markers to predict the efficacy of MBC treatment frequently relate to the characteristics of the primary tumor, even though antigen profiles of the primary tumor and the distant metastases have been reported to differ in 7–20 % of patients [3–7]. Hence, a reassessment of predictive markers, including the expression of human epidermal growth factor receptor 2 (HER2), might help to optimize MBC treatment [8, 9]. Due to the invasive nature of MBC, however, tissue sampling of metastatic sites may be difficult to perform, especially if repeated sampling is required [10].

Tumor cell spread into the blood circulation plays a key role during cancer progression. Currently, highly sensitive methods are being developed to detect single circulating tumor cells (CTCs), which are found in 40–80 % of breast cancer patients with metastatic disease. Using the CellSearch® technology cleared by the United States Food and Drug Administration in 2004, large studies have clearly demonstrated the adverse prognostic impact of CTC counts ≥5 per 7.5 mL peripheral blood in patients with MBC [11–17]. In addition, detection of CTCs in primary breast cancer has been shown to be an independent prognostic marker for survival [18].

Apart from CTC enumeration to estimate prognosis, CTC phenotype determination might be useful in predicting the efficacy of targeted therapy [8, 19–23]. Sampling of metastatic tissue is associated with increased morbidity, limiting the feasibility of repeated analysis. Therefore, CTC characterization may offer an attractive means of noninvasively monitoring the expression of therapeutic targets in patients with breast cancer. In this context, we conducted the present study to compare the expression of HER2 in primary tumor tissue, metastatic tissue, and CTCs. Moreover, we aimed to analyze the impact of HER2 overexpression of CTCs on prognosis.

## Methods

### Patients and study design

Patients treated for MBC at the National Center for Tumor Diseases (NCT; Heidelberg, Germany) between March 2010 and October 2013 were evaluated for this exploratory study. CTC enumeration was performed before starting a new line of systemic treatment. Patients with CTC counts ≥5 CTCs/7.5 mL peripheral blood were defined as CTC-positive [11]. Only CTC-positive patients were included in this analysis. Additional criteria for inclusion were age >18 years, clinical and radiological evidence of measurable or evaluable metastatic disease according to the Response Evaluation Criteria in Solid Tumors (RECIST) criteria [24], progressive metastatic disease, and written informed consent for study participation, data collection and analysis, and publication. Patient records were reviewed for reports of metastatic tissue biopsies. Patients with malignancies other than breast cancer were excluded. Ethical approval was obtained from the Ethics Committee of the Medical Faculty of the University of Heidelberg.

### Enumeration and HER2 characterization of CTCs

Enrichment and enumeration of CTCs using the CellSearch technology (CellSearch™ Epithelial Cell Kit/CellSpotter™ Analyzer, Janssen Diagnostics LLC, Raritan, NJ, USA) was essentially performed as described elsewhere [25]. Briefly, 7.5 mL samples of peripheral whole blood were collected in CellSave tubes (Janssen Diagnostics LLC, Raritan, NJ, USA) containing ethylenediaminetetraacetic acid (EDTA) and a cellular preservative. Samples were maintained at room temperature and processed within 96 h. Epithelial cells were immunomagnetically enriched using ferrofluid nanoparticles coated with antibodies against epithelial cell adhesion molecule (EpCAM). Subsequently, EpCAM-positive cells were labeled with the nuclear dye 4′,6-diamidino-2-phenylindole (DAPI) and immunostained with monoclonal antibodies specific for keratins and for the leukocyte common antigen CD45. Cells with intact nuclei that were CD45-negative and keratin-positive were defined as CTCs and enumerated by trained operators. Blood samples containing ≥5 CTCs/7.5 mL blood were considered CTC-positive, as published previously [11].

HER2 expression on CTCs was characterized within the CellSearch technology using an anti-HER2 antibody labeled with fluorescein isothiocyanate (FITC, CellSearch tumor phenotyping reagent HER2, Janssen Diagnostics LLC, Raritan, NJ, USA), as described previously [19, 26, 27]. The intensity of HER2-specific immunofluorescence was scored as negative (0), weak (1+), moderate (2+), or strong (3+). CTC status was considered HER2-positive (CTC-HER2-positive) if at least one CTC exhibited strong (3+) or moderate (2+) HER2 staining [8].

### Primary and metastatic HER2 status

The HER2 status was determined for the primary tumor (PRIM-HER2 status) and metastatic tissue (MET-HER2 status) using the immunohistochemistry-based HERCEP™ test (DAKO, Glostrup, Denmark) for semi-quantitative detection of HER2 expression in breast cancer tissue. Expression of HER2 was scored on a scale from 0 to 3+. Primary tumor and metastatic tissue samples with a score of 3+ were considered PRIM-HER2-positive and MET-HER2-positive, respectively. In cases where the score was 2+, HER2 amplification was determined by fluorescence in-situ hybridization (FISH) using the Pathvysion Kit (Vysis Inc., Downers Grove, IL, USA).

### Data collection and analysis

All data were extracted systematically from treatment records. Demographic data and clinical characteristics were described as frequency and percentage, median and range, or mean and standard deviation. Groups were compared using the Wilcoxon rank test or Fisher’s exact test, as appropriate. Kaplan–Meier plots by CTC-HER2 status were generated with R, version 3.0.0 [28], for PFS and OS (time from initiation of the new line of systemic treatment to disease progression and death from any cause, respectively), with data being censored at last follow-up if progression or death had not occurred. PFS and OS times were estimated as medians with 95 % confidence intervals (CIs). Differences in PFS and OS by CTC-HER2 status were assessed by the log-rank test. All statistical tests were done using R, version 3.0.0 with package *survival* [28]. A significance level of 5 % was chosen.

## Results

### Patient characteristics

**Fig. 1:**
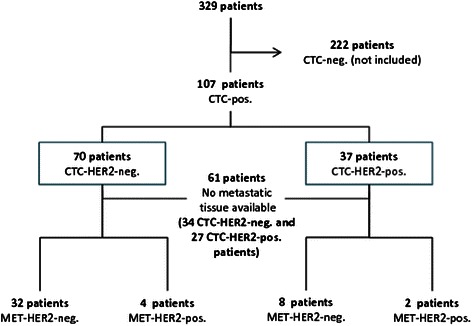
Patient flow through the study

In total, 107 CTC-positive patients with a median age of 57 years (range, 33–81) were included in the analysis. 100/107 (93 %) patients were followed-up for a median [95 % CI] of 28.5 [25.1–40.1] months. Table 1 details the patient characteristics. Figure 1 shows the flow of patients through the study and indicates the number of patients with metastatic tumor tissue. The primary tumor was estrogen receptor (ER)-positive in 78 (73 %) patients, progesterone receptor (PR)-positive in 68 (64 %) patients, and HER2-negative in 91 (85 %) patients. 80 % of all patients had multiple metastatic sites, 18 % had bone metastases, 21 % had visceral or local metastases, and 62 % had both. 48 % of all patients received first-line treatment for MBC, 21 % second-line treatment, and 31 % third- or further-line treatment (line unknown in one patient). 13 % of patients were pretreated with HER2-targeted therapy before study entry. The median time from biopsy of the primary tumor to biopsy of metastatic lesions was 44 months. The median time from biopsy of metastases to CTC analysis was 230 days.

**Table 1:** Patient characteristics and CTC-HER status

	Total	CTC-HER2-positive	*p*-value
Total, *n* (%)	107	37 (35)	
Age at primary diagnosis, years; median (range)	49 (33–81)	49 (35–77)	0.594
Age at enrollment, years; median (range)	57 (33–81)	58 (40–77)	0.517
ER status, *n* (%)			0.253
Negative	78	30 (38)	
Positive	29	7 (24)	
PR status, *n* (%)			0.673
Negative	68	25 (37)	
Positive	39	12 (31)	
Number of metastatic sites, *n* (%)			0.799
One site	21	8 (38)	
Multiple sites	86	29 (34)	
Site of metastasis, n (%)			0.626
Bone	19	5 (26)	
Visceral	22	7 (32)	
Both	66	25 (38)	
Line of therapy, n (%)^a^			0.268
First	51	18 (35)	
Second	22	10 (45)	
Further	33	8 (24)	

*ER* estrogen receptor, *PR* progesterone receptor

^a^Line of therapy unknown for one patient

### HER2 status of CTCs, primary tumor, and metastases

The median number (range) of CTCs detected per 7.5 mL blood was 27 (5–5000). HER2-positive CTCs were detected in 37/107 (35 %) patients. There was no significant association between CTC-HER2 status and CTC count or any other factor shown in Table 1.

As shown in Table 2, only 10 (27 %) of the 37 CTC-HER2-positive patients had a HER2-positive primary tumor (PRIM). The overall accuracy between CTC-HER2 and PRIM-HER2 status was 69 %. The HER2 status of metastatic tissue samples (MET) was available for 46 patients, of whom 6 (13 %) were MET-HER2-positive. HER2-positive metastasis was observed only in 2 out of 10 (20 %) CTC-HER2-positive patients. The overall accuracy between CTC-HER2 and MET-HER2 status was 74 %. As shown in Table 3, the overall accuracy between PRIM-HER2 and MET-HER2 status was 38/46 (83 %). 3/46 (7 %) breast cancers were PRIM-HER2-positive and MET-HER2-negative, whereas 5/46 (11 %) were PRIM-HER2-negative and MET-HER2-positive.

**Table 2:** Comparison of CTCs, primary tumor, and metastatic tissue by HER2 status in patients with MBC

	Total	CTC-HER2-negative	CTC-HER2-positive
		*n* (%)	*n* (%)
PRIM-HER2 status (total)	107	70 (65 %)	37 (35 %)
Negative	91	64 (70 %)	27 (30 %)
Positive	16	6 (38 %)	10 (62 %)
MET-HER2 status (total)	46	36 (78 %)	10 (22 %)
Negative	40	32 (80 %)	8 (20 %)
Positive	6	4 (67 %)	2 (33 %)

Overall accuracy of CTC-HER2 and PRIM-HER2 status: 69 %; overall accuracy of CTC-HER2 and MET-HER2 status: 74 %

**Table 3:** Comparison of primary tumor and metastatic tissue by HER2 status (PRIM-HER2 and MET-HER2 status, respectively)

	Total	MET-HER2-negative	MET-HER2-positive
PRIM-HER2 status (total)	46 (100 %)	40/46 (87 %)	6/46 (13 %)
Negative	42/46 (91 %)	37/42 (88 %)	5/42 (12 %)
Positive	4/46 (9 %)	3/4 (7 %)	1/4 (25 %)

Overall accuracy of MET-HER2 and PRIM-HER2 status: 83 %

### Analysis of survival by CTC-HER2 status

**Fig. 2:**
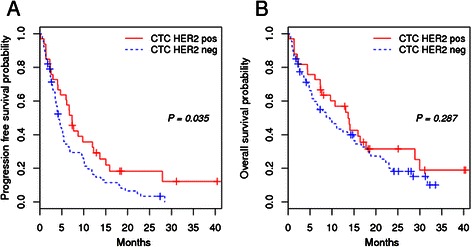
Kaplan–Meier plots of progression-free survival (**a**) and overall survival (**b**) of CTC-positive (≥5 CTCs/7.5 mL blood) MBC patients by CTC-HER2 status

Follow-up data were available for 100/107 (93 %) patients with a median follow-up period [95 % CI] of 28.5 [25.1–40.1] months. Figure 2 shows the Kaplan–Meier plots of PFS and OS by CTC-HER2 status. PFS was significantly longer in CTC-HER2-positive patients than in CTC-HER2-negative patients (*p* = 0.035), the respective median PFS times [95 % CI] being 7.4 [4.7–13.7] and 4.3 [3.5–5.9] months. In contrast, the association of CTC-HER2-positive status with a longer OS of 13.7 [7.7–30.0] months as compared with 8.7 [5.9–15.3] months for CTC-HER2-negative status was not statistically significant (*p* = 0.287).

## Discussion

Although the expression of therapeutic targets may change during the course of disease, treatment decisions for MBC are often based on primary tumor characteristics [29]. Therefore, current treatment guidelines recommend reevaluation of patients with MBC for the HER2 and hormone receptor status of metastatic tissue (www.ago-online.de). However, this is an invasive procedure that could be difficult to perform, especially if sampling needs to be repeated [10]. By contrast, CTCs provide a very promising, easy to repeat, real-time “liquid biopsy” approach. CTCs offer the advantage that the therapeutic targets they express may more accurately represent the currently most important subpopulation of tumor cells thus potentially making CTCs better predictors of the efficacy of targeted treatments [30].

Our results confirm that HER2 expression in metastatic tissue does not necessarily reflect the phenotype of the primary tumor. The process of tumor progression and distant metastasis is highly selective, involves genetic changes that also affect HER2 status, and mainly, is accompanied by selection of HER2-positive or HER2-negative cells. The 83 % overall accuracy in HER2 status we found between metastatic tissue and primary tumor is in line with other studies, which report overall accuracy rates of 70–95 % [3–6]. About 10 % of patients with initially HER2-negative disease presented with HER2-positive metastasis and therefore were potentially exposed to undertreatment in the absence of HER2-directed therapy. Moreover, the HER2 status of a single metastatic biopsy sample might not accurately enough reflect the HER2 status of all metastatic sites [4]. In contrast to tissue sampling from a single metastasis, the detection and enumeration of CTCs represents an attractive, noninvasive alternative that also enables repeated evaluation of the phenotype of the currently circulating, i.e. probably most active, tumor cells [9]. We found a 74 % overall accuracy in HER2 status between metastatic tissue and CTCs. However, this comparison is limited by the fact that the biopsies of metastatic tissue and blood sampling for CTC studies were not performed simultaneously. Ideally, the CTC-HER2 and MET-HER2 assessments should be performed in temporal proximity because CTC status is known to be dynamic and therefore the time between the two determinations may be relevant. However, in routine clinical practice the time lapse can be considerable. Moreover, HER2-targeted treatment of PRIM-HER2-positive patients before biopsy of metastatic tissue or CTC sampling may interfere with HER2 testing. In our present study, metastatic tissue was available only from 10 CTC-HER2-positive patients, and therefore our findings regarding the incidence of HER2-positive metastasis in CTC-HER2-positive patients should be interpreted with caution. Clarification of these aspects requires prospective studies such as the German DETECT study (NCT01619111). This is a phase III multicenter trial in which patients with a HER2-negative primary tumor but HER2-positive CTCs are randomized to standard treatment alone or in combination with the dual HER1/HER2 tyrosine kinase inhibitor lapatinib.

To determine the HER2 status of CTCs, we used an immunofluorescence staining score previously established by Meng et al. [19] and Riethdorf et al. [27]. These authors demonstrated a high overall accuracy between immunofluorescence and FISH analysis of single cells. The present study defined a patient as CTC-HER2-positive if she was CTC-positive with ≥5 CTCs/7.5 mL blood and had at least one CTC with an immunostaining score of 3+ or 2+. This definition was proposed by Fehm et al., who recently presented a prospective multicenter trial investigating HER2 expression on CTCs in patients with MBC [8]. In line with their study, one-third of our patients had at least one CTC with strong or moderate, i.e. 3+ or 2+, overexpression of HER2, and 31 % of patients had a CTC-HER2 status different from their primary tumor’s HER2 status. Hence, it is possible that 10 % of all patients with MBC might benefit from HER2-directed treatment despite their primary tumor being HER2-negative since about 80 % of all patients are PRIM-HER2-negative, half of whom are CTC-positive (≥5 CTCs/7.5 mL blood) in the metastatic setting where the rate of CTC-HER2 positivity (i.e., immunostaining score 3+ or 2+) is about 30 %.

However, there are also other definitions of CTC-HER2 positivity. For instance, Pestrin et al. and Meng et al. defined a patient as CTC-HER2-positive if the receptor was strongly overexpressed on at least 50 % of CTCs [19, 20]. On the other hand, studying primary breast cancer, where the CTC detection rate is lower than in MBC, Riethdorf et al. also used the presence of at least one HER2-positive CTC as a cut-off level [27]. To establish a robust predictive marker, an optimal cut-off level for CTC-HER2 positivity needs to be defined in prospective trials. An even more restrictive definition of CTC-HER2 positivity (only CTCs with 3+ staining) than the one we used in the present study (3+ or 2+ staining) is currently being evaluated in the above-mentioned DETECT study.

Interestingly, we found that patients with HER2-positive CTCs had had a significantly longer PFS than patients who were CTC-HER2-negative. This is in contrast to other studies that evaluated the impact of HER2-positive CTCs on survival [21, 31]. However, these studies compared the prognosis of CTC-HER2-positive patients with the prognosis of patients without CTCs. By contrast, all patients in our study were CTC-positive, which per se is associated with an extremely poor prognosis. However, in view of the relatively small number of patients and the lack of significant impact of CTC-HER2 status on OS, our results should be interpreted with caution. Moreover, these results are likely to be biased by HER-targeted treatment as 11 of the 37 CTC-HER2-positive patients had a HER2-positive primary tumor or HER2-positve metastasis, or both.

### Limitations of the study

Potential limitations of the study include its retrospective design, the lack of FISH diagnostics for all HER2 assessments, the variability of HER2 immunohistochemistry testing, and the fact that the sample size was too small for multivariate analysis.

## Conclusions

Our findings confirm that the HER2 status of breast cancer may change during the course of disease, with important consequences for the efficacy of targeted treatment. In this context, CTC phenotyping may serve as a “liquid biopsy” that is easily performed and repeated during breast cancer treatment and is a promising aid in guiding treatment decisions. The clinical value of CTCs in predicting the efficacy of HER2-directed therapy is currently being investigated in prospective randomized trials.

## Abbreviations

CI: Confidence interval
CTC: Circulating tumor cell
CTC-HER2 status: HER2 status of CTCs
DAPI: 4′,6-diamidino-2-phenylindole
EDTA: Ethylenediaminetetraacetic acid
EpCAM: Epithelial cell adhesion molecule
ER: Estrogen receptor
FISH: Fluorescence in situ hybridization
FITC: Fluorescein isothiocyanate
HER2: Human epidermal growth factor receptor 2
MET-HER2 status: HER2 status of metastatic tissue
NCT: National Center for Tumor Diseases, Heidelberg, Germany
PR: Progesterone receptor
PRIM-HER2 status: HER2 status of primary tumor
RECIST: Response Evaluation Criteria in Solid Tumors
